# Activation-induced Markers Detect Vaccine-Specific CD4^+^ T Cell Responses Not Measured by Assays Conventionally Used in Clinical Trials

**DOI:** 10.3390/vaccines6030050

**Published:** 2018-07-31

**Authors:** Georgina Bowyer, Tommy Rampling, Jonathan Powlson, Richard Morter, Daniel Wright, Adrian V.S. Hill, Katie J. Ewer

**Affiliations:** The Jenner Institute, University of Oxford, Oxford OX3 7DQ, UK; t.rampling@ucl.ac.uk (T.R.); jpowlson@gmail.com (J.P.); richard.morter@ndm.ox.ac.uk (R.M.); danny.wright@ndm.ox.ac.uk (D.W.); adrian.hill@ndm.ox.ac.uk (A.V.S.H.); katie.ewer@ndm.ox.ac.uk (K.J.E.)

**Keywords:** vaccines, activation-induced markers, antigen-specific T cells, viral vector, adenovirus, modified vaccinia virus Ankara, Ebola

## Abstract

Immunogenicity of T cell-inducing vaccines, such as viral vectors or DNA vaccines and Bacillus Calmette-Guérin (BCG), are frequently assessed by cytokine-based approaches. While these are sensitive methods that have shown correlates of protection in various vaccine studies, they only identify a small proportion of the vaccine-specific T cell response. Responses to vaccination are likely to be heterogeneous, particularly when comparing prime and boost or assessing vaccine performance across diverse populations. Activation-induced markers (AIM) can provide a broader view of the total antigen-specific T cell response to enable a more comprehensive evaluation of vaccine immunogenicity. We tested an AIM assay for the detection of vaccine-specific CD4^+^ and CD8^+^ T cell responses in healthy UK adults vaccinated with viral vectored Ebola vaccine candidates, ChAd3-EBO-Z and MVA-EBO-Z. We used the markers, CD25, CD134 (OX40), CD274 (PDL1), and CD107a, to sensitively identify vaccine-responsive T cells. We compared the use of OX40^+^CD25^+^ and OX40^+^PDL1^+^ in CD4^+^ T cells and OX40^+^CD25^+^ and CD25^+^CD107a^+^ in CD8^+^ T cells for their sensitivity, specificity, and associations with other measures of vaccine immunogenicity. We show that activation-induced markers can be used as an additional method of demonstrating vaccine immunogenicity, providing a broader picture of the global T cell response to vaccination.

## 1. Introduction

The majority of currently licensed vaccines mediate protection by inducing antibody responses [1]. Immunogenicity of these vaccines is most often measured by enzyme-linked immunosorbent assays (ELISAs) that measure total antigen-specific antibody titers or assays that measure antibody function. These well-established vaccination programs often have gold-standard assays with a threshold value known to provide clinical protection [2]. Many vaccines currently being developed target pathogens for which neutralizing antibodies are difficult to produce (e.g., HIV) [3,4], that have multiple serotypes (staphylococci and streptococci) [5], or are intracellular (tuberculosis (TB), liver-stage malaria) [6,7]. Additionally, many new vaccines are being developed to target non-communicable diseases, such as autoimmunity and cancer [8,9]. It may be necessary for vaccines against many of these targets to induce potent T cell responses instead of, or in addition to, humoral responses to elicit protection [10]. This new generation of vaccines requires a set of assays that effectively capture antigen-specific T cell responses.

A number of assays are conventionally used to measure the quantity and quality of antigen-specific T cells in humans [11]. The assays most frequently used for this purpose in clinical vaccine trials are the enzyme-linked immunospot (ELISpot) and intracellular cytokine staining (ICS) assays [12,13,14]. The ELISpot assay involves stimulating peripheral blood mononucleocyte (PBMC) with antigen in 96-well plates with membranes coated in the anti-cytokine capture antibody. Cytokines produced by antigen-specific T cells binds to the capture antibody and are detected using an enzyme-conjugated secondary antibody and a chromogenic development substrate. This method is highly sensitive (cells producing fewer than 100 cytokine molecules can be detected [15]) and the lower limit of detection can be under ten cytokine-producing cells per million [16]. ELISpots are currently one of the most frequently used and highly validated assays for the detection of antigen-specific T cell responses in clinical trials [16,17,18]. The major disadvantages of using this assay to investigate vaccine responses are a limit in the number of parameters than can be investigated, lack of phenotypic information, and preferential detection of effector cells. This likely results in an underestimation of the total antigen-specific T cell response. An alternative type of assay that is frequently used to provide further information on the quantity and quality of vaccine-induced T cells is ICS. Antigen-stimulated PBMC are stained with fluorescently labelled anti-cytokine antibodies and analyzed by flow cytometry. This allows detailed phenotypic and functional analysis of antigen-specific T cell populations.

However, ICS assays are also limited in the number of parameters that can be assessed and these must be pre-determined, therefore, these assays can be biased towards the detection of a particular type of T cell. Standard panels in clinical trials often use IFNγ, IL2, and TNFα and, therefore, detect Th1-biased responses [19,20,21]. T cell responses to vaccination and infection can be highly heterogeneous and, therefore, detection based on the expression of one or more cytokines may significantly underestimate the frequency of antigen-specific cells [22,23]. Multiple groups have overcome these limitations by developing T cell assays that define antigen-specificity based on the upregulation of TCR-stimulated surface markers—termed activation-induced markers (AIM). These include an assay based on the detection of OX40 and CD25 co-expression on CD4^+^ T cells in whole blood [24] or PBMC [25,26], and assays detecting CD40L [27,28], or co-expression of CD40L and CD69 [29]. Similarly, activation-induced markers, such as CD107a and CD137 (4-1BB), have been used to identify antigen-specific CD8^+^ T cells [30,31]. Additional markers of activation, such as OX40 and CD25 are expressed on activated CD8^+^ T cells and could be used in a similar way [32]. The advantages of these assays over conventional methods are that they do not rely on prior knowledge of the epitope or HLA type and they are not limited by pre-determination of the cytokine/s to be analysed.

AIM assays can provide a broader picture of the overall antigen-specific T cell response. Increasing knowledge about the total quantity and quality of these responses could significantly aid clinical development, regulatory approval and licensure applications for vaccine candidates [10]. AIM assays have been used to detect vaccine-specific T cell responses in humans [25,33], and we routinely use CD107a as a marker of antigen-specific degranulation in our clinical trials [34,35,36]. However, AIM assays have not been fully evaluated and compared with cytokine-based methods for their use within the clinical trial setting. In this study, we investigated the use of AIM assays to detect antigen-specific CD4^+^ and CD8^+^ T cell responses to vaccination. We then compared the sensitivity and specificity to assays we currently use to investigate vaccine immunogenicity—an IFNγ ELISpot and ICS assay measuring the frequencies of CD4^+^ and CD8^+^ T cells producing IFNγ, IL2, and TNFα, or expressing CD107a. AIM assays sensitively and specifically detected antigen-responsive CD4^+^ and CD8^+^ T cells after vaccination. AIM assays had a comparable (CD4^+^) or lower (CD8^+^) background and detected higher levels of antigen-specific T cells than cytokine-based methods. Additionally, the frequency of AIM^+^ CD4^+^ T cells did not correlate with the frequency of cytokine-production measured by ICS or ELISpot, suggesting that the AIM assay can provide novel information about the antigen-specific CD4^+^ T cell response that is not detected by conventional clinical trial methods.

## 2. Materials and Methods

### 2.1. Clinical Vaccine Trial

The samples used in this study were collected as part of a Phase Ia clinical vaccine trial assessing the safety and immunogenicity of viral vectored Ebola vaccine candidates (EBL04, ClinicalTrials.gov NCT02451891, EudraCT number: 2015-000593-35). The recombinant chimpanzee adenovirus type 3 vectored Ebola Zaire vaccine (ChAd3-EBO-Z) is a replication-deficient chimpanzee adenovirus serotype 3 (ChAd3) vector expressing wild-type (WT) Zaire strain Ebola glycoprotein (GP). The modified vaccinia Ankara virus vectored Ebola Zaire vaccine (MVA-EBO-Z) is a replication-deficient, attenuated vaccinia Ankara virus vector expressing the wild-type Ebola glycoprotein (GP) of the Zaire Mayinga strain.

This study uses samples from all 16 volunteers from Group 2 of the EBL04 study. These individuals received 3.6 × 10^10^ viral particles (vp) ChAd3-EBO-Z and were boosted with 1.0 × 10^8^ plaque forming units (PFU) MVA-EBO-Z seven days later. All vaccinations were administered intramuscularly into the deltoid region of the arm. All volunteers were healthy adults between the ages of 18 and 50 years recruited at the Centre for Clinical Vaccinology and Tropical Medicine at the University of Oxford and the Wellcome Trust Clinical Research Facility at the Imperial College, London, both in the United Kingdom.

### 2.2. Ethics and Regulatory Approval

All participants provided written informed consent prior to enrolment. The study was conducted according to the principles of the Declaration of Helsinki (2008) and the International Conference on Harmonization Good Clinical Practice guidelines. The study protocol and associated documents for the Phase Ia trial were reviewed and approved by the UK National Research Ethics Service (Committee South Central-Oxford A, reference 15/SC/0108, IRAS project ID 174444, approval date: 25/02/2015) and the Medicines and Healthcare Products Regulatory Agency (Ref.: 21584/0341/001-0001, approval date: 24/04/2015). Further details of the study are provided in the clinical trial protocol available with the clinical trial report (Venkatraman et al. manuscript in preparation).

### 2.3. Sample Handling

Blood was collected in heparin-treated vacutainers for peripheral blood mononucleocyte (PBMC) separation and processed within six hours of blood draw to ensure sample quality. All processing was conducted under sterile conditions in a class II microbiological safety cabinet. PBMCs were separated by density gradient isolation using Lymphoprep^®^ (STEMCELL Technologies, Vancouver, BC, Canada) in Leucosep^®^ (Sigma-Aldrich, Saint Louis, MO, USA) tubes. Washes were conducted using R0 medium (RPMI-1640 medium with 5% l-Glutamine and 5% Penicillin/Streptomycin). Cell culture and assays were performed using R10 medium (RPMI-1640 medium, 5% l-Glutamine, 5% Penicillin/Streptomycin and 10% Fetal Calf Serum (FCS)).

Ex vivo IFNγ ELISpot assays and ICS were performed using freshly separated PBMC. AIM assays were conducted using thawed cryopreserved PBMC. PBMC remaining after ELISpot and ICS set up were frozen at 6–10 × 10^6^ cells/mL in FCS with 10% dimethyl sulfoxide (DMSO) with controlled cooling using CoolCell^®^ Cell freezing containers (BioCision, San Rafael, CA, USA) and stored in liquid nitrogen until use. For AIM assays, cryopreserved cells were thawed in a 37 °C water bath. Cells were washed in pre-warmed R10, then resuspended in 2 mL R10 with 5 µL benzonase nuclease (250 units/μL, ≥90%, Novagen, Darmstadt, Germany). Cells were rested at 37 °C and 5% CO_2_ for 2 h before washing and resuspension in R10 to the concentration required for the assay.

### 2.4. IFNγ ELISpot

Ex vivo (18 h stimulation) ELISpot assays were performed using Multiscreen IP ELISpot plates (Millipore), human IFNγ SA-ALP antibody kits (Mabtech, Stockholm, Sweden), and BCIP NBT-plus chromogenic substrate (Moss Inc., Pasadena, MD, USA). Cells were cultured in R10 and antigens were tested in triplicate. To measure GP-specific responses, we used 187 peptides covering the length of the Ebola Zaire glycoprotein (Mayinga strain). GP peptides were mostly 15 amino acids in length, overlapping by 11 amino acids. The sequences were provided by the National Institutes of Health (NIH), USA and are the same as those used in a previous study [37]. There were seven pools consisting of peptides from the glycoprotein chain 1 (GP1, amino acids 33–501) and two pools containing peptides from the glycoprotein chain 2 (GP2, amino acids 504–676). Each peptide pool was added to the plate at a final concentration of 2.5 µg/mL in 150 µL with 200,000 PBMC. A mixture of Staphylococcal enterotoxin B (SEB, 0.02 μg/mL) and phytohaemmagglutinin-L (PHA, 10 μg/mL) was used as a positive control in all ELISpot assays. Negative control wells with no peptide were included in all assays to measure the background. Peptides used in the ELISpot, ICS, and AIM assays were resuspended in DMSO. Negative controls did not include DMSO. However, we have shown in previous experiments that DMSO only causes increases in the background when the concentration is >0.1% (unpublished data). In all of the experiments run here, the peptide pools had final DMSO concentrations of 0.0012% or lower. Plates were developed the following day according to the manufacturer’s instructions. Plates were counted using an automated ELISpot counter (AID Diagnostika (Strausberg, Germany) with identical settings for all plates. Counts were only adjusted to remove artefacts. The average spot forming cells per million PBMC (SFC/10^6^ PBMC) was calculated for each pool on the plate. Responses in unstimulated (negative control) wells were subtracted and then responses in individual pools were summed to give a total response against Ebola GP. ELISpot QC criteria were >800 SFC/10^6^ PBMC in the positive control wells and <80 SFC/10^6^ in the negative control wells. Responses greater than the median + 2 standard deviations of the negative control wells were considered positive. The lower limit of detection for this assay is defined as the lowest number of spots that is precisely distinguishable from the unstimulated control well and is calculated as the median plus 2 standard deviations of all unstimulated control wells run for that study. The detection limit for this study was 50 SFC/10^6^ PBMC.

### 2.5. Intracellular Cytokine Staining (ICS)

Stimulations were set up for ICS in parallel with the ELISpot, using freshly isolated PBMC. Cells were stimulated with a single megapool of the same GP peptides used in the ELISpot, at a final concentration of 2.5 µg/mL. An unstimulated condition and a positive control stimulated with 1 µg/mL SEB were run for each sample. Each stimulation was set up in a 5 mL polystyrene FACS tube containing 2 × 10^6^ PBMC in 1 mL of R10. Anti-CD28 and anti-CD49d (eBioscience, San Diego, CA, USA) at final concentrations of 1 µg/mL and 2 µL of αCD107a-PE-Cy5 (eBioH4A3, eBioscience) were added to each tube. Samples were stimulated for 18 h at 37 °C and 5% CO_2_. Brefeldin A and Monensin (eBioscience), both at 1 μg/mL, were added for the last 16 h. Responses were assessed by a nine-colour staining panel. Cells were incubated for 20 min at room temperature with a dead cell discrimination dye (AQUA 1:2000, Invitrogen) diluted in FACS buffer (PBS with 1% BSA and 0.1% NaN_3_). PBMC were permeabilized using fixation/permeablization solution (BD Biosciences), then stained intracellularly at room temperature (RT) for 30 min with the following antibodies: αCD3-AF700 (UCHT1, 1/1000, eBioscience), αCD4-APC (RPA-T4, 1/500, eBioscience), αCD8-APC-AF780 (RPA-T8, 1/200, eBioscience), αCD14-eF450 (61D3, 1/1000, eBioscience), αCD19-eF450 (HIB19, 1/1000, eBioscience), αIFNγ-FITC (4S.B3, 1/2000, eBioscience), αIL2-PE (MQ1-17H12, 1/1000, eBioscience), and αTNFα-PE-Cy7 (Mab11, 1/10,000, eBioscience). Cells were then washed and fixed in 1% paraformaldehyde. Compensation was performed using single-stained One-Comp beads (eBioscience) for monoclonal antibodies and ARC beads for AQUA (Life Technologies, Carlsbad, CA, USA). Acquisition was performed on the day of staining on a BD LSRII and FACSDiva v6.2 (BD Biosciences, San Jose, CA, USA). Data was prepared and analysis performed using FlowJo v10.1 (Treestar Inc., Ashland, OR, USA). Cells were gated on lymphocytes, singlets, live CD3^+^, CD8^-^CD4^+^, or CD4^-^CD8^+^ and then IFNγ, IL2, TNFα and CD107a. Dead cells (AQUA^+^), monocytes (CD14^+^), and B cells (CD19^+^) were excluded from the analysis. The ICS gating strategy (Supplementary Figure S1) is the same as previously published [38]. Responses to peptide were determined after subtraction of the response in the unstimulated control for each sample. Responses were considered positive if the count was >20 and frequency greater than two times the autologous unstimulated control and greater than the lower limit of detection (LLOD). The LLOD was calculated as the reciprocal of the minimum number of CD4^+^ (21,971) and CD8^+^ (21,528) T cells collected. This resulted in LLOD values of 0.005% for both CD4^+^ and CD8^+^ populations for this dataset. Samples failed quality control and were excluded from data analysis if the background-subtracted response of cytokine positive CD4^+^ or CD8^+^ T cells to the positive control was <1%. In this study 3/16 of the samples either did not have ICS results or did not pass the QC criteria. Therefore the final ICS dataset for this assay included responses from 13 individuals.

### 2.6. Activation-Induced Markers (AIM) Assay

The activation-induced markers assay was conducted in a similar way to previously published work [25,26,39]. PBMC were stimulated overnight (20 h at 37 °C) with 1–2 × 10^6^ cells per well in a 96-well U-bottom plate. A stimulation time of 20 h was chosen because this was used in previous studies and longer incubations were shown to be optimal for OX40 expression [39,40]. Additionally, the clinical trial ICS SOP we use is a 16–20 h stimulation and having incubations of the same length would allow the assays to be easily run in parallel in clinical trials if required. Despite this longer incubation, we did not see evidence of bystander activation. It has been demonstrated that bystander activation only had a minimal effect on the AIM assay despite a 20 h incubation [39]. Cells were stimulated with 2 µg/mL of the same megapool of Ebola GP peptides used for the ICS assay. An unstimulated well and a positive control well stimulated with 1 µg/mL SEB were included for each sample. Cells were stained with αCD107a-PE-Cy5 (eBioH4A3, 1/2000, eBioscience) during the stimulation. After overnight stimulation, cells were washed twice in FACS buffer (PBS with 1% BSA and 0.1% NaN_3_), then stained for 20 min at RT with 100 µL per well of a mixture containing the following fluorescently conjugated antibodies: αCD45RA-eV605 (HI100, 1/200, eBioscience), αCD14-eF506 (61D3, 1/200, eBioscience), αCD19-eF506 (HIB19, 1/200, eBioscience), αCD4-APC-eF780 (RPA-T4, 1/100, eBioscience), αCD3-AF700 (UCHT1, 1/133, eBioscience), αOX40-PE/Cy7 (Ber-ACT35, 1/100, Biolegend, San Diego, CA, USA), αCD25-FITC (M-A251, 1/100, Biolegend), αPDL1-APC (B7-H1, 1/133, Biolegend), αCD8-BV711 (RPA-T8, 1/400, Biolegend), and AQUA live/dead stain (1/2000, Invitrogen, Carlsbad, CA, USA). Cells were washed twice as before, fixed with 4% paraformaldehyde for 10 min at 4 °C, washed twice again, resuspended in 100 µL FACS buffer, and acquired immediately on a BD LSRII. Data analysis was conducted in FlowJo version 10.1 (Treestar Inc.). The LLODs for the assay were determined in the same way as the LLODs for the ICS assay (minimum number of events collected were CD3: 87213, CD4: 32730 and CD8: 19172). The LLODs were 0.001% for total T cells, 0.003% for CD4^+^ T cells, and 0.005% for CD8^+^ T cells. Compensation was performed as for the ICS assay.

### 2.7. Statistical Analysis

The data were non-parametric, therefore, Mann-Whitney analyses were used for comparison between two groups and Kruskal-Wallis analyses with Dunn’s post-tests were used for comparison across multiple groups. Linear regression analyses were conducted using Spearman’s rank. Where responses were compared at two different time points within a group, Wilcoxon matched pairs analyses were used. An alpha-level of 0.05 was considered significant and all *p*-values are 2-tailed. Analyses were performed in GraphPad Prism version 7. (GraphPad Software Inc., La Jolla, CA, USA). Medians and inter-quartile ranges (IQR) are presented. * *p*< 0.05, ** *p* < 0.01, *** *p* < 0.001, **** *p* < 0.0001.

## 3. Results

### 3.1. Detection of Vaccine-Specific T cells Using Activation-Induced Markers

**Figure 1:**
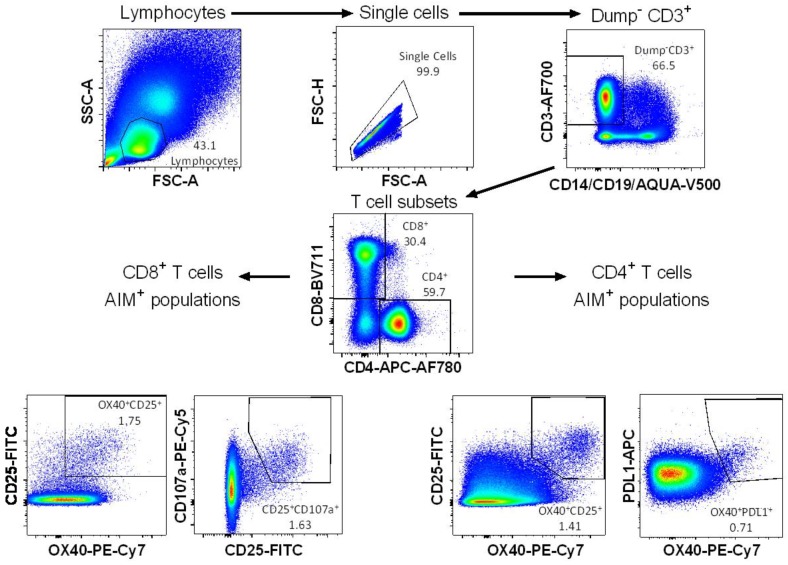
Activation-induced markers (AIM) gating strategy. Cells were gated on single lymphocytes based on size, then dead cells, CD14^+^, and CD19^+^ cells were excluded. T cell subsets were gated as CD4^+^CD8^-^ or CD8^+^CD4^-^ and then the expression of activation-induced markers was measured within each subset. Gates displayed are representative of the top quartile of Ebola glycoprotein (GP)-specific responses to clearly demonstrate where these populations sit.

The expression of combinations of activation-induced markers on CD4^+^ (OX40^+^CD25^+^ and OX40^+^PDL1^+^) and CD8^+^ (OX40^+^CD25^+^ and CD25^+^CD107a^+^) T cells were assessed by flow cytometry using the gating strategy defined in Figure 1.

**Figure 2:**
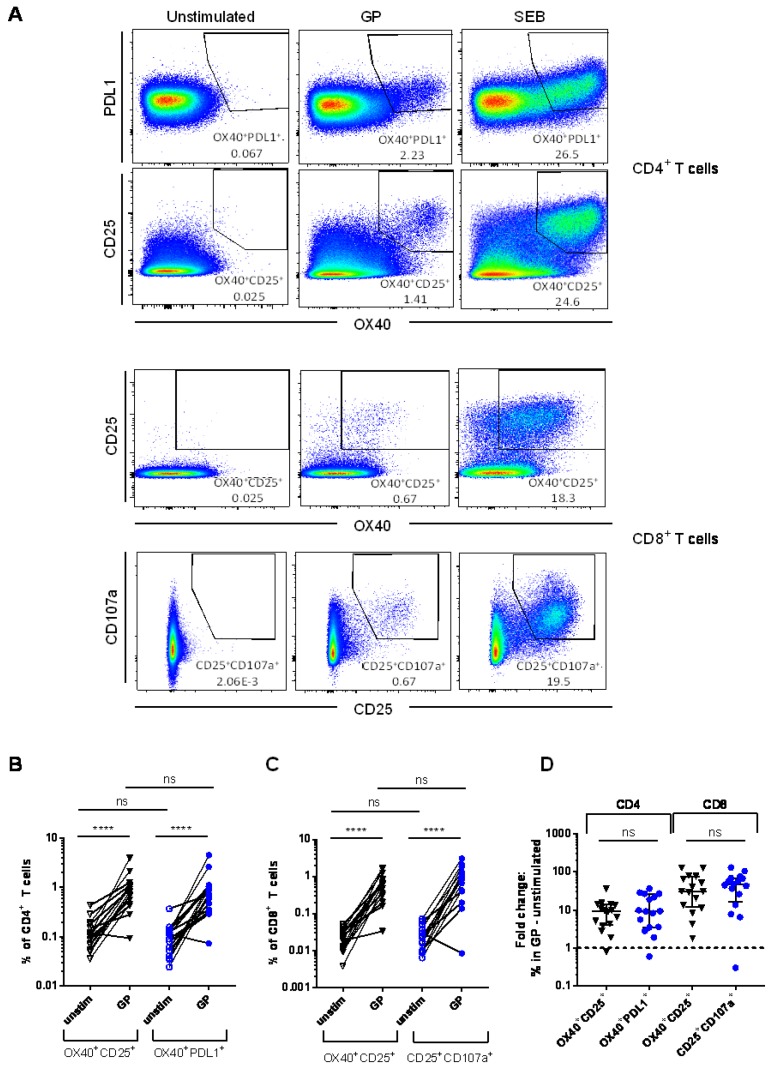
Detection of vaccine antigen-specific T cells: (**A**) Representative flow cytometry plots detailing AIM^+^ populations in unstimulated, GP-stimulated and Staphylococcal enterotoxin B (SEB)-stimulated CD4^+^ and CD8^+^ T cells; (**B**) AIM^+^ responses in CD4^+^ T cells; and (**C**) AIM^+^ responses in CD8^+^ T cells. Mann-Whitney analyses between stimulation conditions within each population and between the same stimulation conditions in different populations. Medians and inter-quartile range (IQR) shown. **** *p* < 0.0001, ns: Not significant (*p* > 0.05); (**D**) fold change in frequency of AIM^+^ cells (GP-stimulated/unstimulated conditions). Individuals below the dashed line did not have responses greater than the background.

Very little CD107a expression was detected in CD4^+^ T cells and PDL1 expression on CD8^+^ T cells was also low, therefore these markers were not included in the analysis of antigen-specific CD4^+^ and CD8^+^ T cell responses, respectively. Vaccine-specific T cell responses could clearly be detected in the CD4^+^ T cell subset as OX40^+^CD25^+^ or OX40^+^PDL1^+^ and in the CD8^+^ T cell subset as OX40^+^CD25^+^ or CD25^+^CD107a^+^. For each sample, an unstimulated control was run to determine background AIM expression and an SEB-stimulated positive control was included. Representative FACS plots of AIM^+^ populations in each condition are shown in Figure 2A.

Frequencies of AIM expression in GP-stimulated PBMC were significantly higher than the corresponding background for all four of the AIM populations measured (Figure 2B,C, *p* < 0.0001 for all populations). Within the CD4^+^ T cell subset, background levels of AIM expression in unstimulated cells were generally low and were comparable between the OX40^+^CD25^+^ and OX40^+^PDL1^+^ populations (Figure 2B, median + inter-quartile range (IQR) OX40^+^CD25^+^: 0.110% (0.069–0.172) and OX40^+^PDL1^+^: 0.102% (0.044–0.131), *p* = 0.468). The background was also low in the CD8^+^ subset and comparable between the two AIM populations (Figure 2C, OX40^+^CD25^+^: 0.021% (0.010–0.033) and CD25^+^CD107a^+^: 0.020% (0.012–0.036), *p* = 0.934). Frequencies of GP-specific CD4^+^ T cells measured using OX40^+^CD25^+^ or OX40^+^PDL1^+^ were comparable (Figure 2B, OX40^+^CD25^+^: 0.870% (0.493–1.088) and OX40^+^PDL1^+^: 0.736% (0.389–1.088), *p* = 0.773). Similar frequencies of GP-specific CD8^+^ T cells were detected and were also comparable for the two different AIM populations in this subset (Figure 2C, OX40^+^CD25^+^: 0.633% (0.319–0.837) and CD25^+^CD107a^+^: 0.882% (0.406–1.258), *p* = 0.224). Due to the lower background in the CD8^+^ subset, the fold-change in the frequency of AIM^+^ cells (GP-stimulated/unstimulated) was higher for the CD8^+^ subset than the CD4^+^ subset (Figure 2D, OX40^+^CD25^+^ CD4^+^: 9 (4–14), OX40^+^PDL1^+^ CD4^+^: 9 (4–26), OX40^+^CD25^+^ CD8^+^: 31 (12–73), CD25^+^CD107a^+^ CD8^+^: 47 (17–68)). However, there was no difference between the marker combinations in either of the subsets (CD4^+^: *p* = 0.662, CD8^+^: *p* = 0.616).

### 3.2. Comparison of Different Activation-Induced Markers for Detection of Vaccine-Specific T Cells

**Figure 3:**
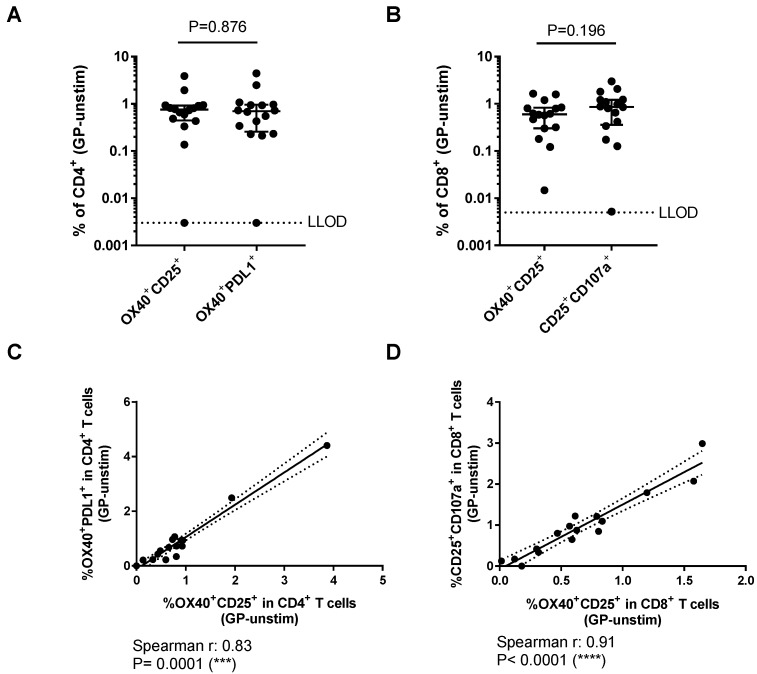
Comparison of activation-induced markers for the detection of Ebola GP-specific T cells: (**A**) Proportion of AIM^+^ cells in GP-stimulated CD4^+^ and; (**B**) CD8^+^ subsets after removal of background (frequency in unstimulated condition). Dashed lines indicate the lower limit of detection (LLOD). Mann-Whitney analyses used for comparisons between markers; (**C**) relationship between the frequency of GP-specific CD4^+^ T cells measured by OX40^+^CD25^+^ and OX40^+^PDL1^+^; and (**D**) relationship between the frequency of GP-specific CD8^+^ T cells measured by OX40^+^CD25^+^ and CD25^+^CD107a^+^.

The frequency of GP-specific T cell responses was compared between the different AIM^+^ subsets after subtracting the corresponding background for each sample (AIM^+^ frequency in the unstimulated condition, Figure 3A,B). Frequencies of OX40^+^CD25^+^ and OX40^+^PDL1^+^ in CD4^+^ T cells were comparable (0.753% (0.445–0.924) and 0.700% (0.259–0.961), respectively, *p* = 0.876). All, but one individual (15/16), had responses above the LLOD (0.003%) in both AIM populations. The frequencies of AIM^+^ cells detected by either of the marker combinations in the CD8^+^ subset were also comparable (OX40^+^CD25^+^: 0.601% (0.304–0.826) and CD25^+^CD107a^+^: 0.861% (0.359–1.219), *p* = 0.196). Almost all vaccines had detectable levels of AIM^+^ CD8^+^ T cells measured by both marker combinations. One individual did not have a CD25^+^CD107a^+^ response above the LLOD (0.005%), although they did have a detectable OX40^+^CD25^+^CD8^+^ population. Frequencies of GP-specific T cells measured by each of the combinations of AIM markers was highly correlated in both CD4^+^ (Figure 3C, Spearman *r* = 0.83, *p* = 0.0001) and CD8^+^ T cells (Figure 3D, Spearman *r* = 0.91, *p* < 0.0001).

### 3.3. Relationship Between AIM Assays and Other Measures of Vaccine Immunogenicity

**Figure 4:**
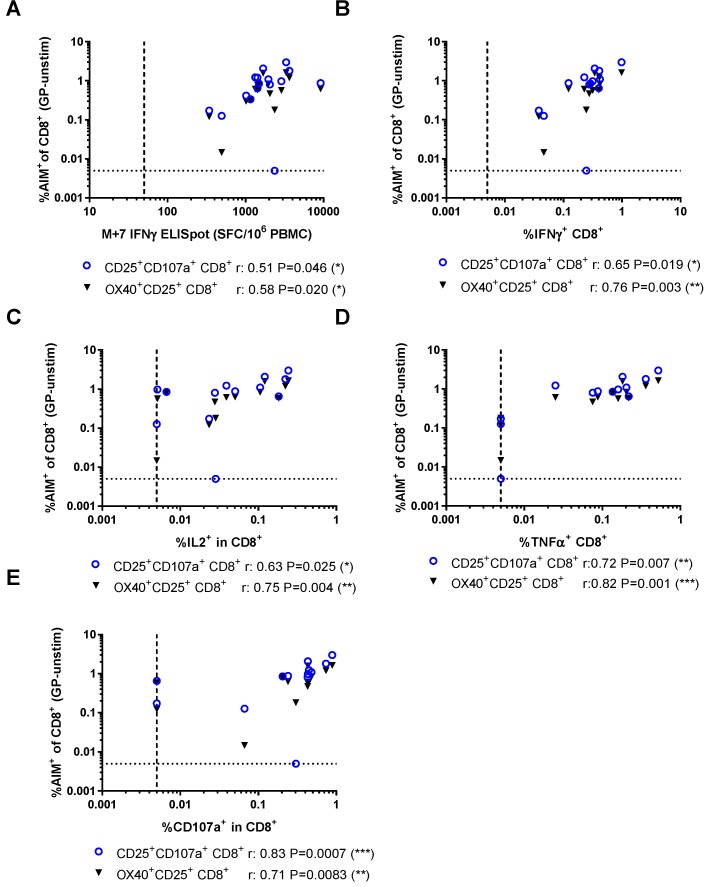
Relationships between activation-induced markers on CD8^+^ T cells and other measures of vaccine immunogenicity: (**A**) IFNγ enzyme-linked immunospot (ELISpot); (**B**) frequency of IFNγ^+^ CD8^+^ T cells measured by intracellular cytokine staining (ICS); (**C**) frequency of IL2^+^ CD8^+^ T cells measured by ICS; (**D**) frequency of TNFα^+^ CD8^+^ T cells measured by ICS; and (**E**) frequency of CD107a^+^ CD8^+^ T cells measured by ICS. SFC/10^6^ PBMC: Spot-forming cells per million peripheral blood mononucleocytes. Spearman’s rank analyses.

**Figure 5:**
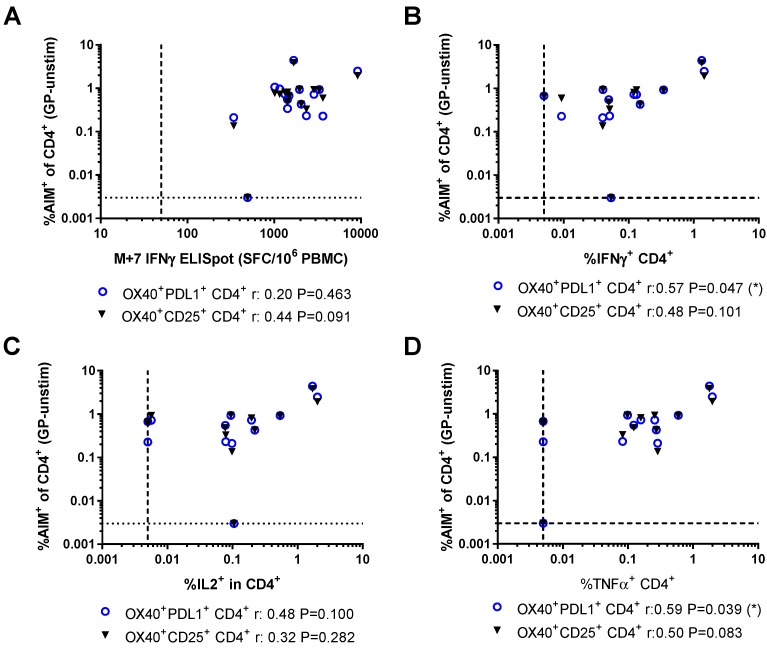
Relationships between activation-induced markers on CD4^+^ T cells and other measures of vaccine immunogenicity: (**A**) IFNγ ELISpot; (**B**) frequency of IFNγ^+^ CD4^+^ T cells measured by ICS; (**C**) frequency of IL2^+^ CD4^+^ T cells measured by ICS; and (**D**) frequency of TNFα^+^ CD4^+^ T cells measured by ICS. SFC/10^6^ PBMC: Spot-forming cells per million peripheral blood mononucleocytes. Spearman’s rank analyses.

The relationships between the frequency of GP-specific T cells detected by the AIM assay and other methods used in the clinical vaccine trial were compared. At the peak time point (seven days after MVA boost, M+7), GP-specific T cell responses were measured using IFNγ ELISpot. Responses were further dissected using ICS to analyse the frequency of CD4^+^ and CD8^+^ T cells producing IFNγ, IL2, and TNFα, or expressing CD107a. Frequencies of AIM^+^ CD8^+^ T cells measured using either combination of markers were correlated with all other methods used to detect GP-specific CD8^+^ T cell responses at this time point (Figure 4). In contrast, the frequency of GP-specific OX40^+^CD25^+^ CD4^+^ T cells did not correlate with any of the other measures of GP-specific CD4^+^ responses and there were only weak associations between GP-specific OX40^+^PDL1^+^ and IFNγ^+^ (Spearman *r* = 0.57, *p* = 0.047) or TNFα^+^ (Spearman *r* = 0.59, *p* = 0.039) CD4^+^ T cells measured by ICS (Figure 5).

**Figure 6:**
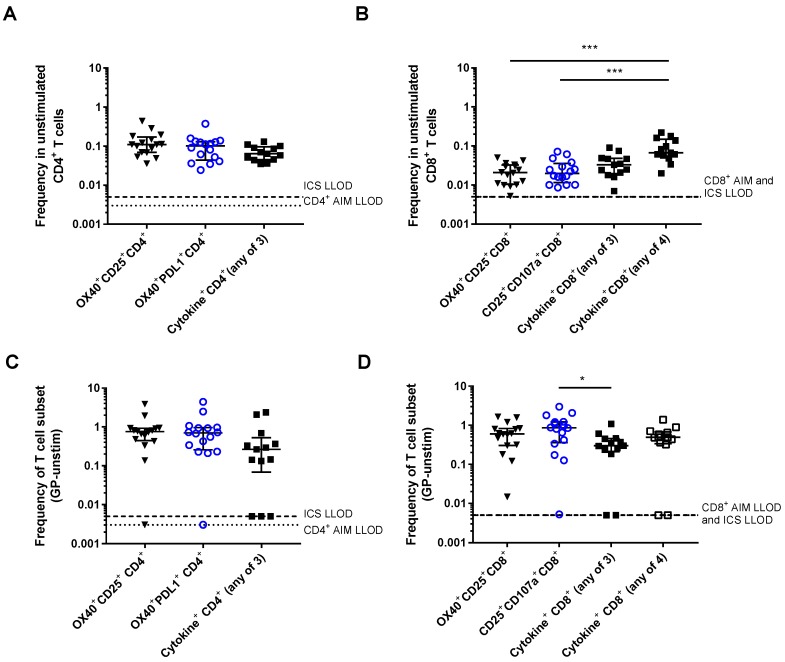
Specificity and sensitivity of the AIM assay compared with trials ICS protocol: (**A**) Frequency of AIM^+^ cells or cells producing at least one of the three cytokines measured (IL2, TNFα, or IFNγ) by ICS in unstimulated CD4^+^ T cells. Kruskal-Wallis *p* =0.092; (**B**) frequency of AIM^+^ cells, cells producing at least one of the three cytokines measured (IL2, TNFα, or IFNγ), or producing/expressing any of the four (IL2, TNFα, IFNγ or CD107a) in unstimulated CD8^+^ T cells. Kruskal-Wallis *p* = 0.0001; (**C**) frequency of GP-specific AIM^+^ cells in CD4^+^ T cell subset producing at least one of the three cytokines measured (IL2, TNFα or IFNγ). Kruskal-Wallis *p* = 0.058; and (**D**) frequency of AIM^+^ cells in CD8^+^ T cell subset, producing at least one of the three cytokines measured (IL2, TNFα, or IFNγ), or producing/expressing any of the four (IL2, TNFα, IFNγ, or CD107a). Kruskal-Wallis *p* = 0.027. Dashed lines show LLOD for ICS assays (0.005 for both CD4^+^ and CD8^+^ T cells), dotted lines show LLOD for AIM assays (0.003 for CD4^+^ and 0.005 for CD8^+^). * *p* < 0.05, *** *p* < 0.001.

The specificity of the AIM assay for detecting GP-specific T cells was compared with the ICS assay (Figure 6A,B). The frequency of AIM expression in unstimulated CD4^+^ T cells was not significantly different to the frequency of cytokine production by unstimulated cells in the ICS assay (Figure 6A, OX40^+^CD25^+^: 0.110 (0.069–0.172), OX40^+^PDL1^+^: 0.102 (0.044–0.131), ICS “any of three”: 0.064 (0.045–0.096), Kruskal-Wallis *p* = 0.092). The frequency of AIM expression in unstimulated CD8^+^ T cells was lower than the frequency of cytokine production in unstimulated CD8^+^ T cells in the ICS assay (Figure 6B, OX40^+^CD25^+^: 0.021 (0.010–0.033), CD25^+^CD107a^+^: 0.020 (0.012–0.036), ICS “any of three”: 0.033 (0.020–0.048), ICS “any of four”: 0.067 (0.053–0.150), Kruskal-Wallis *p* = 0.0001).

The sensitivity of the AIM assay for detecting GP-specific T cells was compared with the ICS assay (Figure 6C,D). Within CD4^+^ T cells, this was defined as the frequency producing any of the three cytokines tested: IFNγ, IL2, or TNFα, “any of three”. Within CD8^+^ T cells, this was defined as either “any of three” or as the frequency producing any of the three cytokines or expressing CD107a, “any of four”. For each of these assays, GP-specific T cell frequency was calculated by subtracting the frequency in the unstimulated condition from that in the GP-stimulated condition. Within the CD4^+^ subset, the LLOD for the AIM assay was lower (0.003%) than for the ICS assay (0.005%) and the frequency of GP-specific CD4^+^ T cells detected was higher (OX40^+^CD25^+^: 0.753% (0.445–0.924), OX40^+^PDL1^+^: 0.700% (0.259–0.961)) than that detected by ICS “any of three” (0.265% (0.069–0.527)). Additionally there were three individuals whose responses were below the LLOD in the ICS assay, whilst there was only one individual whose response was below the LLOD in the AIM assay. In the CD8^+^ subset, the LLOD was the same for both assays (0.005%). Again, the frequency of GP-specific T cells detected by the AIM assay was higher than by the ICS assay (OX40^+^CD25^+^: 0.601% (0.304–0.826), CD25^+^CD107a^+^: 0.861% (0.359–1.219), ICS “any of three”: 0.298% (0.210–0.456), ICS “any of four”: 0.492% (0.341–0.676)). The AIM assay using CD25 and CD107a markers detected a significantly higher frequency of GP-specific CD8^+^ T cells than the ICS “any of three”, but there were no other significant differences across the four measures (Kruskal-Wallis *p* = 0.027).

**Figure 7:**
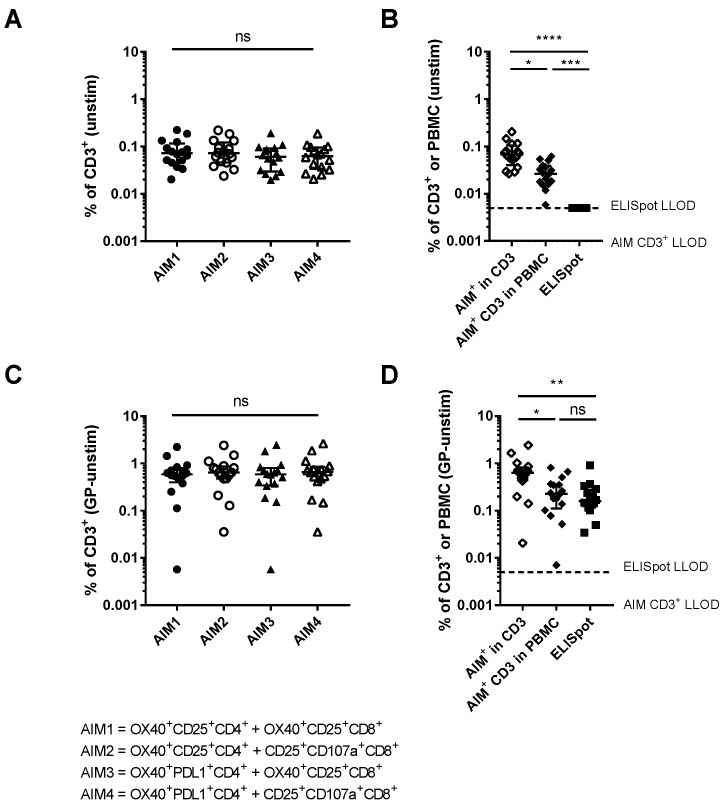
Specificity and sensitivity of AIM assay compared with ELISpot using cells at M+7: (**A**) Frequency of AIM^+^ cells within total T cells using each combination of AIM markers in CD4^+^ and CD8^+^ T cells (Kruskal-Wallis *p* = 0.677). (**B**) Average of the four AIM combinations as a frequency of CD3^+^ T cells and as a frequency of PBMC compared with the background in the ELISpot assay (% IFNγ^+^ PBMC) (Mann-Whitney *p* < 0.0001). (**C**) Frequency of antigen-specific T cells (AIM^+^ in GP-stimulated cells after background subtraction) detected using each of the marker combinations (Kruskal-Wallis *p* = 0.902). (**D**) Average of these combinations as a frequency of CD3^+^ T cells and as a frequency of PBMC compared with the ELISpot (% IFNγ^+^ PBMC) results (Kruskal-Wallis *p* = 0.0018). Dashed lines show LLOD for ELISpot (0.005), dotted lines show LLOD for AIM in total T cells (0.001). ns: No significance, * *p* <0.05, ** *p* <0.01, *** *p* <0.001, **** *p* <0.0001.

AIM^+^ populations within CD4^+^ and CD8^+^ T cells were combined (frequency of AIM^+^ CD4^+^ + AIM^+^ CD8^+^ in CD3^+^) to compare the specificity and sensitivity of AIM and ELISpot assays for detecting the frequency of antigen-specific total T cells (Figure 7). The frequency of AIM^+^ cells within CD3^+^ cells was calculated using each of the combinations of CD4 and CD8 AIM markers and an average taken across the combinations of markers. The frequency of AIM^+^CD3^+^ within PBMC was also calculated for a more direct comparison with the ELISpot assay, for which the input is PBMC and therefore the result is the frequency of IFNγ^+^ PBMC. The LLOD for AIM^+^ total T cells was 0.001%. There were no significant differences in the level of background AIM^+^ T cells between the different marker combinations (Figure 7A, OX40^+^CD25^+^CD4^+^ and OX40^+^CD25^+^CD8^+^: 0.073 (0.047–0.116), OX40^+^CD25^+^CD4^+^ and CD25^+^CD107a^+^CD8^+^: 0.072 (0.046–0.122), OX40^+^PDL1^+^CD4^+^ and OX40^+^CD25^+^CD8^+^: 0.061 (0.030–0.091), OX40^+^PDL1^+^CD4^+^ and CD25^+^CD107a^+^CD8^+^: 0.063 (0.034–0.095), Kruskal-Wallis *p* = 0.677). The average AIM^+^ frequency from these populations was compared with the background in the ELISpot (Figure 7B). The ELISpot results were converted from SFC/10^6^ PBMC to percentages for these analyses. The background level of AIM^+^ T cells was significantly higher than the background in the ELISpot assay, in which no individuals had responses above the LLOD of 50 SFC/10^6^ PBMC or 0.005% (%AIM^+^ in T cells: 0.067 (0.040–0.104), %AIM^+^ T cells in PBMC: 0.026 (0.016–0.039) ELISpot: 0.005, Kruskal-Wallis *p* < 0.0001). The frequency of antigen-specific total T cells (frequency in GP-stimulated after background subtraction) was compared for each the marker combinations and there were no significant differences (Figure 7C OX40^+^CD25^+^CD4^+^ and OX40^+^CD25^+^CD8^+^: 0.593 (0.399–0.804), OX40^+^CD25^+^CD4^+^ and CD25^+^CD107a^+^CD8^+^: 0.641 (0.477–0.866), OX40^+^PDL1^+^CD4^+^ and OX40^+^CD25^+^CD8^+^: 0.590 (0.343–0.803), OX40^+^PDL1^+^CD4^+^ and CD25^+^CD107a^+^CD8^+^: 0.661 (0.434–0.850), Kruskal-Wallis *p* = 0.902). The frequency of antigen-specific cells within total T cells detected by the AIM assay was significantly greater than the antigen-specific response detected by the ELISpot. However the frequency of antigen-specific PBMC detected by each assay was not significantly different (Figure 7D, %AIM^+^ in T cells: 0.633 (0.443–0.820), %AIM^+^ T cells in PBMC: 0.227 (0.111–0.364), %IFNγ^+^ PBMC measured by ELISpot: 0.159 (0.120–0.275), *p* = 0.0009). Comparisons of the AIM and cytokine-based assays are summarized in Table 1.

### 3.4. Activation-Induced Markers as a Measure of Durable Vaccine Responses

**Figure 8:**
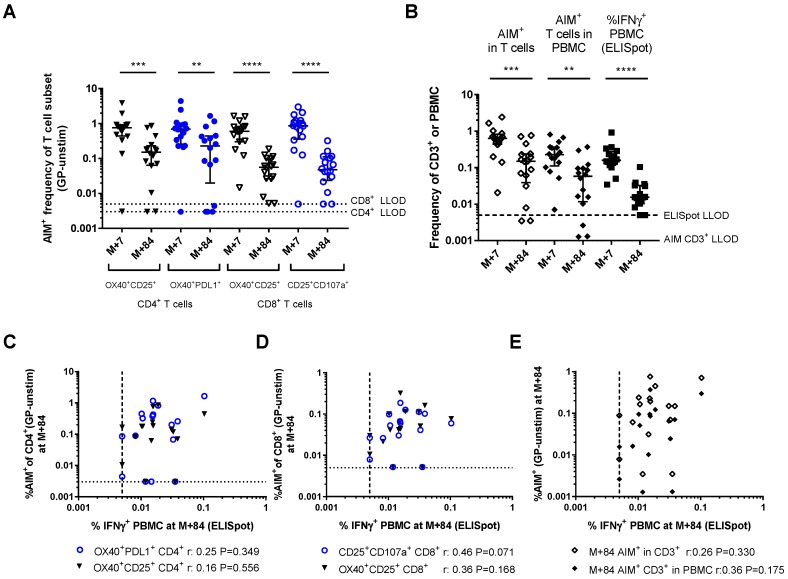
Detection of AIM^+^ T cells at late time points: (**A**) Frequency of AIM^+^ cells within the CD4^+^ and CD8^+^ T cell subsets three months after MVA-EBO-Z vaccination (M+84) compared with peak responses seven days after vaccination (M+7); (**B**) GP-specific T cells measured by ELISpot (%IFNγ^+^ PBMC) or AIM^+^ cells in the total T cells or PBMC at M+7 and M+84. Wilcoxon matched-pairs analyses for comparisons across time points. Relationship between the proportion of GP-specific T cells at M+84 measured by IFNγ ELISpot and; (**C**) frequency of AIM^+^ CD4^+^ T cells at M+84; (**D**) frequency of AIM^+^ CD8^+^ T cells at M+84; and (**E**) frequency of AIM^+^ cells within T cells or AIM^+^ T cells within PBMC at M+84. ** *p* < 0.01, *** *p* < 0.001, **** *p* < 0.0001.

The durability of vaccine responses in this clinical trial were measured by IFNγ ELISpot three months after MVA-EBO-Z vaccination (M+84). The frequency of the GP-specific T cell response detected at this time point by the ELISpot and AIM assays was compared. GP-specific T cell responses measured by all methods were significantly reduced from peak to three months post-boost (Figure 8A,B). However, almost all individuals still had detectable frequencies of AIM^+^ cells within the CD4 and CD8 subsets and total T cells. GP-specific CD8^+^ responses were detectable for 14/16 individuals, whilst GP-specific CD4^+^ responses were detectable for 13/16 individuals by OX40 and PDL1 markers or for 14/16 individuals by OX40 and CD25 markers. At M+84, there was a higher frequency of GP-specific CD4^+^ T cells than CD8^+^ T cells measured by the AIM assay (Figure 8A, OX40^+^CD25^+^CD4^+^: 0.153% (0.064–0.231), OX40^+^PDL1^+^CD4^+^: 0.230% (0.020–0.443), OX40^+^CD25^+^CD8^+^: 0.056% (0.026–0.101), CD25^+^CD107a^+^CD8^+^: 0.048% (0.024–0.112)).

IFNγ ELISpot results at this time point, which include both CD4^+^ and CD8^+^ responses, were ten-fold lower than at the peak (Figure 8B, 0.0155% (0.010–0.032) compared with peak responses of 0.159% (0.120–0.275)). Additionally, 2/16 volunteers did not have detectable ELISpot responses at this time point. In comparison, AIM^+^ cells as a frequency of the total T cells were only four-fold lower than at the peak (M+84: 0.150% (0.039–0.236), M+7: 0.633% (0.443–0.820)) and AIM^+^ T cells as a frequency of PBMC were also four-fold lower than at the peak (M+84: 0.058% (0.012–0.095), M+7: 0.227% (0.111–0.364)). There was no correlation between the AIM assay and ELISpot at this late time point (Figure 8C–E, frequency of AIM^+^ cells in CD4^+^, CD8^+^ or CD3^+^, or frequency of AIM^+^CD3^+^ cells in PBMC).

## 4. Discussion

Antigen-specific T cell responses in clinical trials have classically been determined using cytokine-based approaches, such as ELISpot and ICS assays [12,13,14]. These assays allow for sensitive and specific detection of antigen-responsive T cells without the need for labor-intensive HLA typing. However, these methods require pre-determination of the cytokines to be analyzed, which may cause a bias in the type of T cells detected, and likely underestimate the frequency of the antigen-specific response. Memory cells that are produced in response to both infection and vaccination consist of multiple populations with different gene expression profiles and functional capacity [41,42]. Crucially, these cells differ in their ability to secrete cytokines [43]. In particular, in several studies, it has been observed that the majority of antigen-specific central memory CD4^+^ T cells were not secreting TNFα or IFNγ and, therefore, were not detected by assays based on cytokine secretion [44,45]. Especially at late time points after infection and vaccination, when the ratio of central to effector memory T cells is likely to be higher, the majority of the antigen-specific T cells may be undetectable by cytokine-based assays [46]. Therefore, we investigated the use of activation-induced markers for the analysis of antigen-specific T cell responses to vaccination in a clinical trial and compared the sensitivity and specificity of this method with IFNγ ELISpot and ICS assays.

One possible issue with the AIM assay is the background caused by bystander activation. This has been investigated in a study that found the level of bystander activation to be relatively low [39]. We found that the AIM assay provided a high level of specificity, with background levels comparable or lower than those in the ICS assay, and typically with frequencies lower than 0.15% of the CD4^+^ or CD8^+^ T cell subset. This is lower than, or comparable to, the level of background observed for these and other combinations of AIMs previously investigated [25,30,39].

An issue with the use of OX40 and CD25 markers is the inclusion of antigen-responsive Tregs. This was addressed in a previous study by the addition of PDL1 to discriminate between antigen-responsive Tregs and non-Tregs [39]. In this study by Reiss and colleagues, several marker combinations were directly compared in T cells stimulated with tetanus peptides. In each of these populations, only a minority of the cells were Tregs (based on FOXP3 expression): 1.6% of OX40^+^PDL1^+^ CD4^+^ T cells, 6.8% of CD69^+^CD40L^+^ CD4^+^ T cells, and 16% of the OX40^+^CD25^+^ CD4^+^ T cells. These data suggest that OX40/CD25 are useful markers for identifying the maximal antigen-specific population, whilst OX40/PDL1 or CD69/CD40L can be used to discriminate antigen-specific non-Tregs. In our study, AIM frequencies in GP-stimulated CD4^+^ T cells detected using either of the marker combinations tested were comparable and the two populations were highly correlated. However, this may not be the case for all vaccine antigens at all time points or in all populations. Therefore, an assay including OX40, CD25, and PDL1 could be of use in clinical trials to measure both the maximal antigen-specific response and to discriminate non-Tregs. This may be particularly useful in some circumstances where antigen-specific Tregs are thought to play an important role, such as in malaria-exposed populations [47,48,49].

All of the AIM marker combinations used in our study sensitively detected antigen-specific T cells. The lower limit of detection for the AIM assay in this study was either comparable to (CD8^+^ AIM 0.005%) or lower than (CD4^+^ AIM 0.003%) the ICS assay (0.005% for CD4^+^ and CD8^+^ T cells). Antigen-specific T cells detected using the AIM assay were 2.6–2.8-fold higher than ICS for CD4^+^ T cells and 1.2–1.8-fold higher than ICS for CD8^+^ T cells at the peak time point. Although ELISpot assays are highly specific, the AIM assay tested here was much more sensitive and had a higher signal-to-noise ratio. However it should be noted that the LLOD for the AIM and ICS assays are calculated by the reciprocal of the lowest number of events collected in the appropriate gate (CD4^+^, CD8^+^ or CD3^+^) and will therefore vary depending on the number of cells used in each assay and their viability. In this study 2 × 10^6^ PBMC were used for the ICS, which was conducted as a primary immunological assay in the clinical trial according to an established standard operating procedure. For the AIM assay 1–2 × 10^6^ PBMC were used depending on the number of cryopreserved cells remaining.

These assays capture a comparable level of vaccine-specific T cells in the CD8^+^ subset at the peak time point. However, there was far less agreement between the assays within the CD4^+^ subset, suggesting that we may be missing a substantial portion of the CD4^+^ T cell response to vaccination. This was particularly apparent at late time points. Detection of CD4^+^ T cells and central memory cells not producing cytokine may be an important part of the durability of vaccine responses that may be underestimated. The ELISpot assay is sensitive enough to detect antigen-specific IFNγ-secreting cells in almost all volunteers at least three months after the peak response. ICS is often not performed at these time points in clinical trials as the ability to detect IFNγ-secreting cells by ICS is lower and very few responses are detected [22]. However, the AIM assay results at this late time point indicate that antigen-specific CD4^+^ T cells may be particularly well maintained, an observation that could not be made from the ELISpot assay.

Additionally, a recent study showed that the frequency of antigen-specific CD4^+^ T cells detected by cytokine secretion was significantly reduced (between three and five-fold) after cryopreservation; ICS or ELISpot using thawed cells showed lower and more CD8-biased responses than those using PBMC ex vivo [50]. The ability to perform T cell assays using cryopreserved cells is crucial in many clinical trials—particularly those involving multiple centres. Equally, conducting assays using cryopreserved cells allows samples from multiple time points to be batched, increasing efficiency and reducing experimental variation. Although we have not directly assessed AIM expression on fresh and frozen PBMC, we show here that AIM assays conducted using cryopreserved cells give comparable or higher antigen-specific signals than ICS and ELISpot using PBMC ex vivo.

This study is the first in-depth assessment of AIM assays in CD4^+^ and CD8^+^ T cells in a clinical trial setting. The ICS and ELISpot assays routinely used in clinical trials are highly effective at identifying antigen-specific effector cells at the peak time point. Here we have directly compared the use of AIM and cytokine-based assays for the identification of vaccine-specific T cell responses in clinical trials. There has been substantial work towards standardizing T cell assays for use in human trials and further studies are required to develop and validate AIM assays to a similar level [51,52,53]. These would include studies to optimize the concentration of stimulating antigen, choose which markers to use, and compare responses ex vivo with those from thawed PBMC. Studies are also needed to asses assay variability, particularly between different operators. Additionally it will be important to characterise the functionality of these cells and determine their clinical relevance. For many of the vaccines currently in development, there are no clear immunological correlates of protection in humans. Therefore, determination of protective correlates is an important part of the vaccine development process [2,54,55,56]. The AIM assay is a tool, which in combination with functional measures, could aid the search for immunological correlates of protection. In fact, antigen-specific T cells defined by CD40L expression that were also producing TNFα or IL2 induced by RTS,S/AS vaccination were higher in individuals protected from malaria [57]. However, the markers used in our study have not yet been used to detect antigen-specific T cells in a Phase II vaccine study. It would be useful to include this assay in Phase II clinical studies to determine the clinical relevance of these subsets.

We show that AIM assays can be used to detect antigen-specific T cells with a sensitivity and specificity comparable to or higher than the cytokine-based assays conventionally used in clinical vaccine trials. Additionally, AIM assays may provide novel information about non-cytokine-secreting cells and the durability of the response, particularly in the CD4^+^ subset. AIM assays may provide a more comprehensive account of the total antigen-specific response and, in combination with additional phenotypic and functional markers, could be a valuable tool for more detailed assessments of vaccine-specific responses.

## 5. Conclusions

A combination of AIM assays with traditional cytokine analysis could provide a more complete assessment of the antigen-specific response to vaccination, particularly within CD4^+^ T cells and when assessing durability. Further validation of these assays would be required for use as a primary immunogenicity measure within clinical trials. However, this could significantly enhance knowledge about vaccine-specific T cell responses and aid vaccine development.

## Figures and Tables

**Table 1:** Comparison of sensitivity and specificity of activation-induced markers (AIM) and cytokine-based assays.

Assay/Markers	Lower Limit of Detection (% of Subset)	Frequency in Unstimulated (% of Subset)	Antigen-Specific Signal at M+7 (GP-Unstim, % of Subset)	Antigen-Specific Signal at M+84 (GP-Unstim, % of Subset)
ELISpot	0.005	0.005	0.159 (0.120–0.275)	0.015 (0.010–0.032)
ICS: CD4^+^ “any of three”	0.005	0.064 (0.045–0.096)	0.265 (0.069–0.527)	NA
ICS: CD8^+^ “any of three”	0.005	0.033 (0.020–0.048)	0.298 (0.210–0.456)	NA
ICS: CD8^+^ “any of four”	0.005	0.067 (0.053–0.150)	0.492 (0.341–0.676)	NA
AIM: CD4^+^ OX40^+^CD25^+^	0.003	0.110 (0.069–0.172)	0.753 (0.445–0.924)	0.153 (0.064–0.231)
AIM: CD4^+^ OX40^+^PDL1^+^	0.003	0.102 (0.044–0.131)	0.700 (0.259–0.961)	0.230 (0.020–0.443)
AIM: CD8^+^ OX40^+^CD25^+^	0.005	0.021 (0.010–0.033)	0.601 (0.304–0.826)	0.056 (0.026–0.101)
AIM: CD8^+^ CD25^+^CD107a^+^	0.005	0.020 (0.012–0.036)	0.861 (0.359–1.219)	0.048 (0.024–0.112)
AIM: CD3^+^ (average of marker combinations)	0.001	0.067 (0.040–0.104)	0.633 (0.443–0.820)	0.150 (0.039–0.236)
AIM^+^CD3^+^ (average of marker combinations) in PBMC	0.001	0.026 (0.016–0.039)	0.227 (0.111–0.364)	0.058 (0.012–0.095)

Medians (inter-quartile ranges), NA—not applicable—assay not conducted at this time point.

## Supplementary Materials

The following are available online at http://www.mdpi.com/2076-393X/6/3/50/s1, Figure S1: Intracellular cytokine staining gating strategy. Cells were gated on single lymphocytes based on size. Dead cells, CD14^+^ and CD19^+^ cells were excluded and T cells were identified by CD3 expression. T cell subsets were gated as CD4^+^ and CD8^+^ populations. Cytokine expression was quantified by plotting pairs of cytokines against each other and gating positive populations.
