# Supplementary material for: Activation-induced Markers Detect Vaccine-Specific CD4+ T Cell Responses Not Measured by Assays Conventionally Used in Clinical Trials
Source: Vaccines (Basel). 2018 Jul 31;6(3):50. doi: 10.3390/vaccines6030050 (PMC6161310; doi:10.3390/vaccines6030050)
Supplement: Supplementary file 1 [file vaccines-06-00050-s001.pdf]

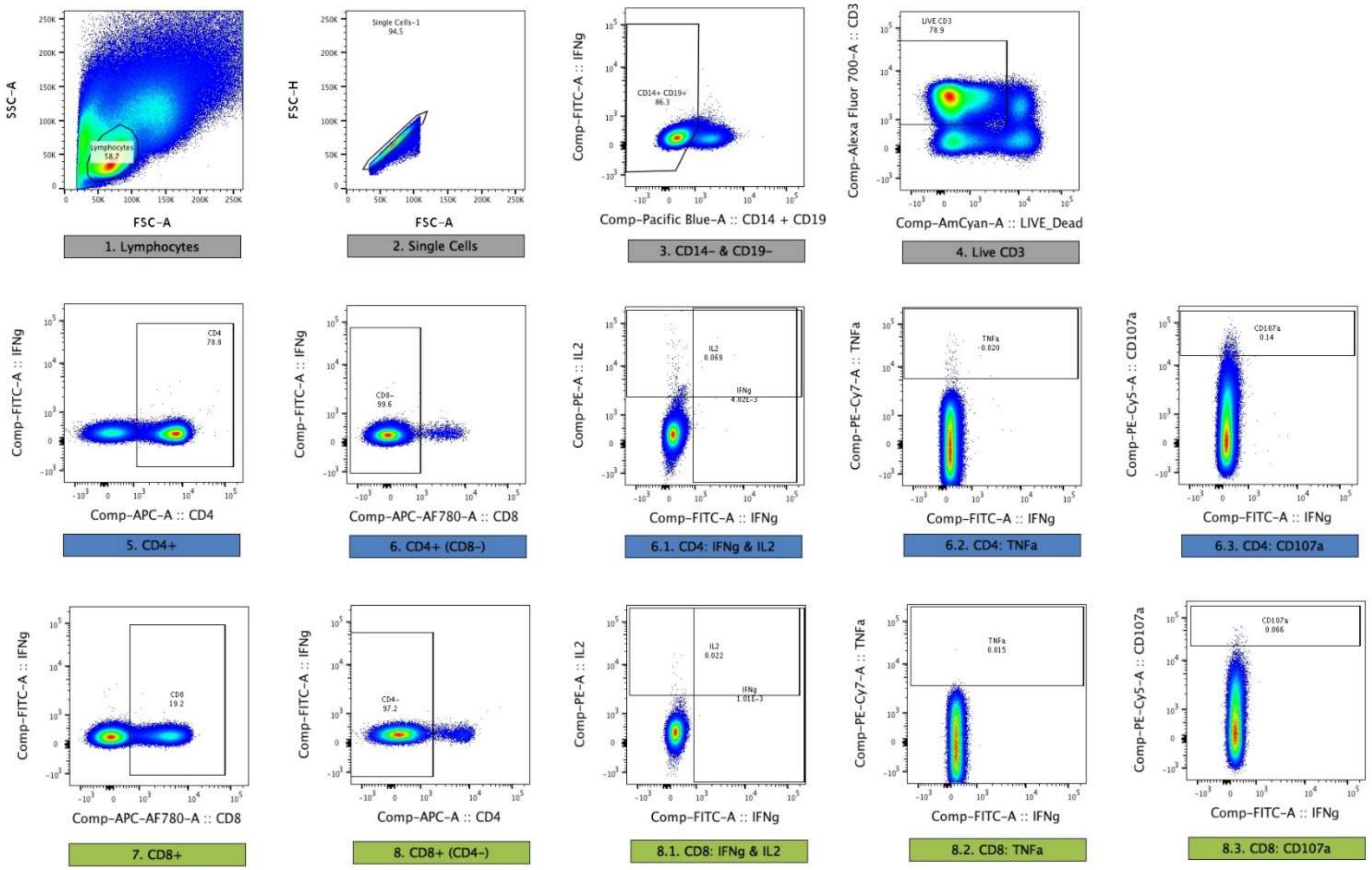

Supplementary Figure S1. Intracellular cytokine staining gating strategy. Cells were gated on single lymphocytes based on size. Dead cells, CD14⁺ and CD19⁺ cells were excluded and T cells were identified by CD3 expression. T cell subsets were gated as CD4⁺ and CD8⁺ populations. Cytokine expression was quantified by plotting pairs of cytokines against each other and gating positive populations.
